# Improved canine exome designs, featuring ncRNAs and increased coverage of protein coding genes

**DOI:** 10.1038/srep12810

**Published:** 2015-08-03

**Authors:** Bart J.G. Broeckx, Christophe Hitte, Frank Coopman, Geert E.C. Verhoeven, Sarah De Keulenaer, Ellen De Meester, Thomas Derrien, Jessica Alfoldi, Kerstin Lindblad-Toh, Tim Bosmans, Ingrid Gielen, Henri Van Bree, Bernadette Van Ryssen, Jimmy H. Saunders, Filip Van Nieuwerburgh, Dieter Deforce

**Affiliations:** 1 Laboratory of Pharmaceutical Biotechnology, Faculty of Pharmaceutical Sciences, Ghent University, Ghent, Belgium; 2 Institut de Génétique et Développement de Rennes, CNRS-URM6290, Université Rennes1, Rennes, France; 3 Department of Applied Biosciences, Faculty of Bioscience Engineering, University College Ghent, Ghent, Belgium; 4 Department of Medical Imaging and Small Animal Orthopaedics, Faculty of Veterinary Medicine, Ghent University, Merelbeke, Belgium; 5 Broad Institute of MIT and Harvard, Cambridge, Massachusetts, USA; 6 Department of Medicine and Clinical Biology of Small Animals, Faculty of Veterinary Medicine, Ghent University, Merelbeke, Belgium; 7 Science for Life Laboratory, Department of Medical Biochemistry and Microbiology, Uppsala University, Uppsala, Sweden

## Abstract

By limiting sequencing to those sequences transcribed as mRNA, whole exome sequencing is a cost-efficient technique often used in disease-association studies. We developed two target enrichment designs based on the recently released annotation of the canine genome: the exome-plus design and the exome-CDS design. The exome-plus design combines the exons of the CanFam 3.1 Ensembl annotation, more recently discovered protein-coding exons and a variety of non-coding RNA regions (microRNAs, long non-coding RNAs and antisense transcripts), leading to a total size of ≈152 Mb. The exome-CDS was designed as a subset of the exome-plus by omitting all 3’ and 5’ untranslated regions. This reduced the size of the exome-CDS to ≈71 Mb. To test the capturing performance, four exome-plus captures were sequenced on a NextSeq 500 with each capture containing four pre-capture pooled, barcoded samples. At an average sequencing depth of 68.3x, 80% of the regions and well over 90% of the targeted base pairs were completely covered at least 5 times with high reproducibility. Based on the performance of the exome-plus, we estimated the performance of the exome-CDS. Overall, these designs provide flexible solutions for a variety of research questions and are likely to be reliable tools in disease studies.

In 2014, the first report detailing the design and performance of a whole exome sequencing (WES) enrichment assay for the dog was published by our group^1^. Aiming to selectively sequence all the regions that are transcribed to mRNA, WES is a reliable tool used to identify disease-causing or predisposing mutations at a fraction of the price of whole genome sequencing (WGS) studies. A limitation of WES is that it is based on our current knowledge of the annotation of the genome and that many disease causing mutations are likely to fall outside protein-coding regions. With new information becoming available, updates and extensions are required. Recently, an improved annotation for the dog genome has been published and new data on non-protein coding genes has been obtained^2^. Based on this data, two new target enrichment designs for dogs, called the exome-plus and the exome-CDS, were developed. The exome-plus offers the most comprehensive design. The exome-CDS is a subset of the exome-plus, focusing on the coding DNA sequences (CDS) by excluding the 3′ and 5′ untranslated regions (UTRs). These two designs offer flexible solutions for a variety of research questions associated with targeted dog exome resequencing. Our current study describes the development of the new designs and the performance of the exome-plus. Based on the results of the exome-plus, we estimate the performance of the exome-CDS. In addition, we provide an in-depth comparison with the previously published exome-1.0^1^.

## Results and Discussion

### Design

Commercially available target enrichment technologies are able to capture up to 200 Mb, which is around 10% of the dog genome. The choice of which regions to include can therefore be based on practical and theoretical considerations, instead of technical limitations. A smaller design does not necessarily result in a cheaper target capture assay, as most commercial custom target capture design tools will increase the tiling density of the baits when the target region size decreases and thus a similar amount of baits are produced. This increased tiling density might increase the capture efficiency. The main cost difference between smaller and bigger designs lies in the increased sequencing cost: more sequence reads will need to be generated to achieve the same sequencing depth on a bigger target compared to a smaller captured region of interest. With these considerations in mind, two separate designs were developed.

The first design, called the exome-plus, has a total size of 151,698,592 bp (≈6% of the genome) divided over 242,914 regions. The exome-plus contains both protein-coding genes and their UTRs and specific non-coding genes. The protein-coding regions contain the exons from the Ensembl annotation (*Canis familiaris*, CanFam 3.1) and newly discovered protein-coding exons recently identified by RNA-sequencing^2^. The non-coding genes are a combination of the microRNAs from miRBase^3^, experimentally characterized long non-coding RNA^2^ and antisense transcripts^2^.

The second design, the exome-CDS, was designed to be a subset of the exome-plus, containing only the CDS from both the Ensembl annotation and the newly discovered protein-coding genes. The 3′ and 5′ UTRs were thus excluded. Candidate CDS within transcript sequences were identified through TransDecoder^4^. Interestingly, this bioinformatics tool discovered a small number of additional exons and CDS, adding a total of 115,044 bp (0.16% of the size exome-CDS) that were not shared with the exome-plus. Overall, the exome-CDS targets 71,254,801 bp (≈3% of the genome) spread over 244,543 regions.

Based on these designs, capturing baits were developed by Roche Nimblegen to target specific regions. When the baits were designed, the regions on the mitochondrial DNA were omitted to avoid overcapturing and oversequencing of the mitochondrial DNA compared with the nuclear DNA^5^. If mitochondrial DNA sequencing is required, one of the options would be to design baits separately and to spike them in at a low concentration.

### Sequencing

In total, 16 canine Labrador Retriever DNA samples were sequenced using the exome-plus design. To assess performance, four separate captures were performed, each consisting of four different pre-capture pooled and indexed samples. Each pool was sequenced in a separate run on a NextSeq 500 Illumina sequencing system. These results were also used to estimate the coverage performance for the exome-CDS, which is a subset of the exome-plus. On average, 243 million reads were generated per sample (Table 1). Following quality trimming, mapping and duplicate reads removal, 87.2% of the reads were retained on average. This result is comparable with previous reports^1^,^6^.

### Performance of the exome-plus: coverage

The on target sequencing depth for the exome-plus varied from 42.6x to 93.9x and was on average 68.3x (Table 1). To assess the regions and base pairs covered, a cut-off sequencing depth of 5x was used as this is the threshold applied usually for variant calling.

**Figure 1:**
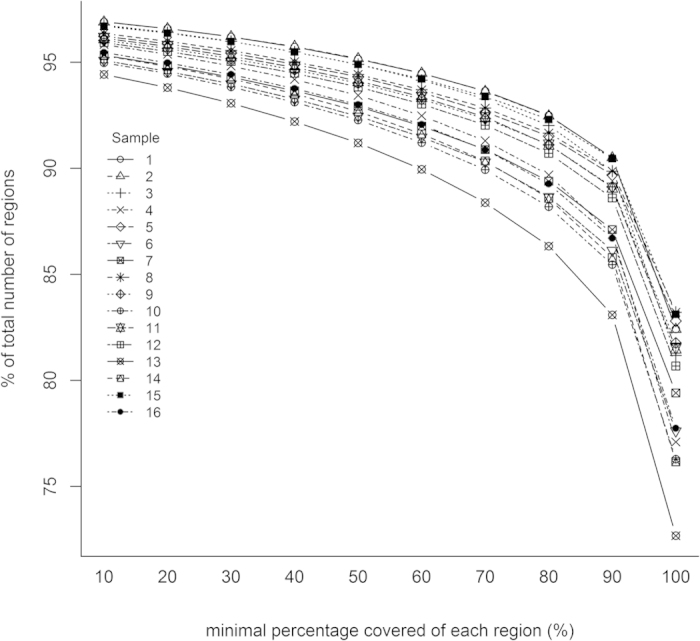
Relation between the minimal percentage covered of each region (%) and the percentage of the total number of regions (%). For each individual region, the proportion of the region covered at a minimum sequencing depth of 5x, was calculated.

From the total of 242,914 targeted regions of the exome-plus, on average 193,722 regions (79.7%) were completely covered with a depth of at least five reads. The number of partially sequenced target regions with a minimal percentage covered, increases when the minimal required percentage of coverage is lowered: e.g. 88% of the regions are covered at least 90%. For on average 9192 regions (3.8%), the maximum sequencing depth reached was four reads. An overview can be found in Table 2. The relation between the number of regions with a minimal coverage and the percentage minimally covered is visualized in Fig. 1.

In terms of covered base pairs, on average 95.1% of the targeted bases pairs reach a minimum sequencing depth of 5 (Table 3). Overall, these results are similar to commercially available human exome sequencing kits^6^.

### Performance of the exome-plus: percentage reads on target

The percentage (%) reads on target is calculated as the number of reads on target, divided by the total number of reads. This parameter informs us of the enrichment efficiency. Overall, the average % reads on target (for all chromosomes and samples) is 75.8%. The lowest average chromosome % reads on target is 63.3% for chromosome X, the highest is 82.2% for chromosome 9. Only a small difference on the % reads on target for chromosome X was noticed when the two sexes were compared: the average % reads on target was 62.6% for males (n = 8) and 63.8% for females (n = 8). Detailed results are provided in Supplementary Table S1. The obtained percentages were similar in the sequenced samples.

### Performance of the exome-plus: reproducibility

We determined the overall reproducibility for both the targeted regions and the targeted base pairs. For all 16 samples, 154,318 regions (63.5%) were completely covered at a minimum sequencing depth of 5x in every single sample. For 4,220 (1.7%) of the regions the maximum sequencing depth reached was four reads for all 16 samples. In terms of base pairs, 137,071,014 base pairs (90.4%) are consistently sequenced at least five times and 3,642,390 base pairs (2.4%) never reach a sequencing depth of 5x.

### Assessment of possible reasons for differences in sequencing depth between regions

**Figure 2:**
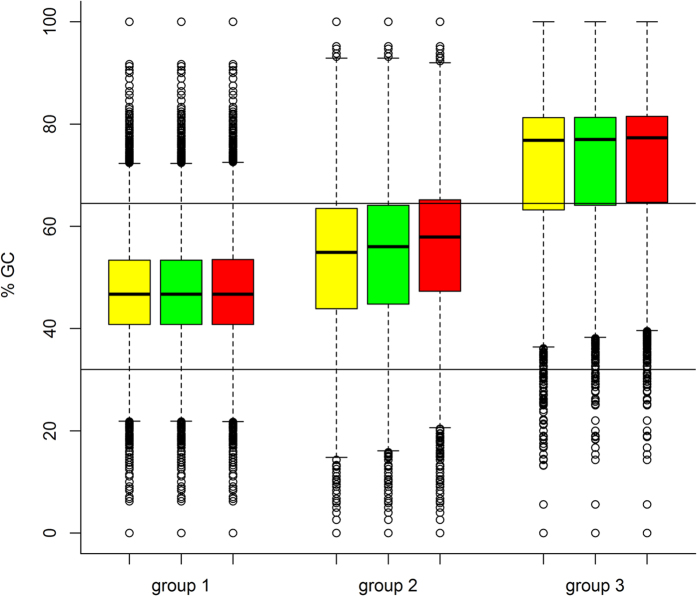
Comparison of the GC content per region (% GC) for the completely covered regions with a minimum sequencing depth of 5x (=group 1), the regions with a varying sequencing depth (=group 2) and the regions with a maximum sequencing depth of 4x (=group 3). Each box represents the 25^th^ (Q1), median (Q2) and the 75^th^ (Q3) quartile, the whiskers represent 1.5 times the interquartile range (Q3–Q1). Outliers are represented as circles. Vertical lines represent the cutoffs at 32.0% GC and 64.5% GC. The yellow boxes represent the values for all the regions in a group. The green boxes represent the values for the remaining regions after exclusion of the regions that Roche Nimblegen predicted to not being sequenced. The red boxes represent the values for the remaining regions after exclusion of all the regions with repeats and Ns.

It seems that the regions can be divided in three categories based on their sequencing performance. Group 1 contains the 154,318 regions that were completely covered in all sixteen samples at a minimum sequencing depth of 5x. Group 2 contains the 84,376 regions that at least partially didn’t reach a minimum sequencing depth of 5x in all 16 samples. The last group, group 3, contains the 4,220 regions with a maximum sequencing depth of 4x for all sixteen samples. We evaluated whether differences in GC content and bait design could be linked to the obtained sequencing performance of these regions. It has been reported that a low sequencing depth can be caused by a high or low GC content^6^,^7^,^8^. Initially, we compared the GC content per region (% GC) for all three groups, resulting in median % GC values of 46.7%, 54.9% and 76.8% for group 1, 2 and 3, respectively (Fig. 2, yellow boxes). Sharp drops of sequencing depth have been reported with % GC above 60.0% and below 40.0%^6^. As all the regions in group 1 were completely covered, we considered that group to be a reliable reference and used the 2.5^th^ and 97.5^th^ percentile of that group as cut-off values for a sequenceable % GC. Based on these cut-offs (which were 32.0% and 64.5%, respectively), we determined for each group the proportion of regions with a more extreme % GC. The proportion of regions with a more extreme value were 4.9%, 24.4% and 75.2% for group 1, group 2 and group 3, respectively. Based on these results, especially group 3 has a relatively large group of extreme % GCs.

Roche Nimblegen does not design baits in regions with low complexity or regions that are highly repetitive to avoid off-target sequencing (called the “repeats” from now on). In addition, for a small number of regions in the canine genome, the exact nucleotide composition is unknown (called the “Ns” from now on), making bait design difficult. Our next step was to determine if any group contained more of these regions. Upon request, Roche Nimblegen provided us with two BED files containing the regions with no baits directly designed for due to repeats and due to Ns, respectively. For group 3, 275 regions (6.5%) contained Ns and 90 regions (2.1%) repeats. Overall, this results in 364 of the regions (8.6%) being (at least partially) excluded for bait design. For group 2, 1,210 regions (1.4%) have Ns and 17,119 regions (20.3%) contained repeats. Overall, this results in 18,136 of the regions (21.5%) being (at least partially) excluded for bait design. For group 1, no regions contained Ns and 1,798 (1.2%) contained repeats. Overall, group 2 contains the largest proportion of regions (partially) excluded from bait design. As some regions in group 1 were consistently sequenced but were at least partially excluded from bait design, it seems that some regions could still be sequenced efficiently due to the presence of neighboring baits. During the designing process, Roche Nimblegen tries to predict this as well and provided an additional BED file that identifies regions that are predicted not to be sequenced. These regions are a subset of the repeat and Ns regions. We compared their estimates with our results and this showed that 0.1%, 7.2% and 1.6% of the regions in group 1, group 2 and group 3, respectively were predicted not to be sequenced by Roche. For group 1, this result seems to be close to correct. Group 2 contained again the largest proportion of difficult regions.

We also compared the % GC of the remaining regions after 1) exclusion of the regions that Roche Nimblegen predicted not to be sequenced and 2) exclusion of all regions that were at least partially excluded from bait design due to Ns and/or repeats (Fig. 2, green and red boxes, respectively). This allows us to check whether the % GC of the remaining regions in each group differs from the overall % GC in each group. For group 1, the median % GC of 46.7% remained identical and the proportion of regions with an extreme % GC remained nearly the same (from 4.9% over 4.9% to 5.0%). For group 2, the median % GC increased from 54.9% over 56.0% to 57.9%. The proportion of regions with an extreme % GC increased likewise from 24.4% over 26.0% to 29.5%. For group 3, the median value and proportion of regions with an extreme % GC only increased slightly (from 76.8% over 77.0% to 77.3% and 75.2% over 75.4% to 76.0%, respectively). It seems that in the second group, the remaining regions tend to have slightly higher % GCs, which might negatively influence sequencing.

In the end, we combined the criteria for the % GC (with 32.0% and 64.5% as cut-offs) and the bait design results to determine the total proportion of regions in each group considered to be at risk for reduced sequencing. Due to extreme % GC and/or regions excluded due to Ns/repeats, 6.1%, 44.6% and 78.1% of the regions for group 1, group 2 and group 3, respectively, were identified to be at risk for reduced performance. Due to extreme % GC and/or regions that were predicted not to be sequenced, 5.0%, 31.4% and 75.8% of the regions for group 1, group 2 and group 3, respectively, were identified to be at risk.

Overall, our criteria seem to be relatively correct as they classify the largest proportion of regions at risk in group 3, the second largest in group 2 and only a small amount in group 1. Specifically for group 3, the majority of the regions seems to be insufficiently covered due to extreme % GC. For group 2, the results seem to be a more balanced combination of extreme % GC and bait design.

### Estimating the coverage for the exome-CDS

Although all samples were sequenced with the exome-plus, we believe we can reliably estimate the performance of the exome-CDS with respect to coverage and reproducibility. This is due to the fact that the exome-CDS is (almost entirely) a subset of the exome-plus. We might underestimate the performance a little bit for the exome-CDS due to the constant number of target baits in each capturing assay. Each target enrichment sequencing assay contains 2.1 million baits. As the exome-CDS is half the size of the exon-plus, twice the number of baits can be used per region. Taking these considerations into account, the following coverage results might be conservative. From the 244,543 regions in the exome-CDS, on average 208,950 regions (85.4%) are estimated to have a sequencing depth of at least 5x throughout the entire region. For on average 9,031 regions (3.7%), the maximum sequencing depth estimated was 4x. In terms of base pairs, we estimate that on average 93.4% (66,586,495 base pairs) of the targeted base pairs are sequenced at least five times. As for the reproducibility, we estimated that in all 16 samples 174,667 regions (71.4%) would be completely sequenced at a minimal sequencing depth of 5x and for 4138 regions (1.7%) the maximum sequencing depth reached would be 4x. In terms of base pairs, 62,455,013 (87.7%) of the base pairs were estimated to be covered consistently in all samples and 2,438,559 (3.4%) base pairs consistently not. The % on target of the exome-CDS was not assessed as a part of the off-target reads for the exome-CDS would actually be on-target reads based on the exome-plus. Including these in the calculation would lead to an underestimation of the % on target.

### Variant calling

As WES is often used in disease-association studies, variant detection is an essential part^9^. Overall, between 250,196 and 278,688 non-reference variants were detected inside the targeted regions of the exome-plus (Table 4). Filtering for those variants inside the exome-CDS, reduces this number to 110,047 to 122,429 variants (Table 4).

### Comparison with the exome-1.0: design

**Figure 3:**
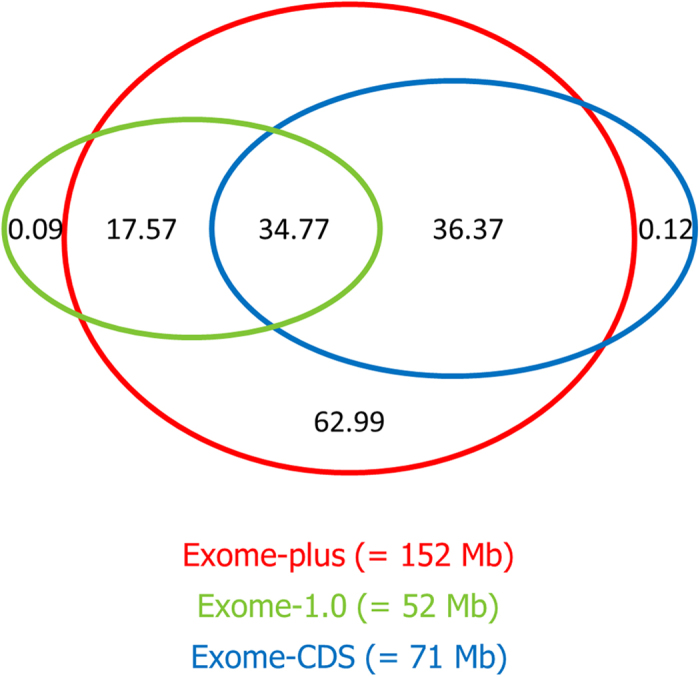
Venn diagram showing the overlap between the exome-1.0 (=52 Mb), exome-CDS (=71 Mb) and the exome-plus (=152 Mb). The depicted numbers represent the size in Mb for the various intersections. Overall, 34.77 Mb is shared by all designs. Inside the target space of the exome-plus, the exome-1.0 targets 17.57 Mb more than the exome-CDS and the exome-CDS targets 36.37 Mb more than the exome-1.0. Finally, 0.09 Mb, 0.12 Mb and 62.99 Mb are targeted uniquely by the exome-1.0, the exome-CDS and the exome-plus, respectively.

A visual comparison between the exome-plus, the exome-CDS and the exome-1.0 can be found in Fig. 3. Overall, 34.77 Mb are shared between all three designs. Although the vast majority is targeted by the exome-plus, a small number of base pairs is targeted uniquely by the exome-1.0 (0.09 Mb) and the exome-CDS (0.12 Mb). The difference between the exome-1.0 and exome-plus is attributable to a small number of genes not being shared by Ensembl Genes and the RefSeq Genes and/or mRNA database. The difference between the exome-CDS and the exome-plus is caused by a small number of additional exons and CDS identified by TransDecoder, as described in the design section. Inside the target space of the exome-plus (152 Mb), the exome-plus and the exome-CDS contain respectively 62.99 Mb and 36.37 Mb more compared with the exome-1.0. For the exome-plus, these differences are attributable to the inclusion of all the new protein-coding genes and the non-protein coding regions that were not available when the exome-1.0 was designed. For the exome-CDS, this difference is smaller due to the exclusion of UTRs from the newly discovered proteins. Finally, besides the 0.09 Mb already mentioned, the exome-1.0 contains an additional 17.57 Mb that is not shared with the exome-CDS. This difference is caused by the exclusion of the UTRs from the Ensembl Genes in the exome-CDS. These UTRs are incorporated in the exome-1.0.

### Comparison with the exome-1.0: performance

An overall comparison of the average performance parameters of the exome-plus, the exome-CDS and the exome-1.0 can be found in table 5. The exome-plus has the lowest scores for the completely covered regions and the region reproducibility. This is attributable to the average size of each individual region. For the exome-plus, ≈152 Mb is divided over 242,914 regions, leading to an average size of each region of 624 base pairs. For the exome-1.0, similar calculations lead to an average region size of only 260 nucleotides. If for even one nucleotide in a region, a sequencing depth of 5x is not reached, this region is not covered completely. Theoretically, we can assume that the probability for this to happen is much more likely when the region size increases. This is in agreement with the experimental results: we divided the target regions in those with a length <260 and ≥260 bp. Next, we compared in each subgroup the proportion of regions that were completely covered, with the total number of regions in this subgroup. On average, 25.0% more regions were completely sequenced if the region size was under 260 bp.

The exome-plus scores highest in terms of base pairs covered and base pair reproducibility. At the same time, the % reads on target is lower in the exome-plus compared with the exome-1.0. These results are explained by the settings applied when the baits were designed. For the exome-1.0, only unique baits were allowed, i.e. baits that only match one location. This is in contrast with the exome-plus that allowed up to 20 matches for each bait. This increases the number of target regions and target base pairs being sequenced at the expense of a lower % reads on target.

The contrasting results of regions and base pairs are due to the combination of the increased region size and the “more matches allowed” bait design settings. Overall, this leads to more regions (and base pairs) being covered for a relatively large proportion, but not completely (Fig. 1). Compared with the exome-1.0, the exome-plus covers 0.9% regions more for 90%^1^.

### Intended use and user-specific customization options

With the development of the exome-plus and the exome-CDS, three WES enrichment assays are available for use. The exome-1.0 contains the core set of protein coding genes. As both the exome-plus and the exome-CDS contain many regulatory regions, they are especially valuable in complex disease studies where mutations influencing expression are more likely to be involved^2^. The exome-plus is the design of choice when one needs the most comprehensive capture based on the most recent annotation of the dog genome, including virtually all transcribed regions. The exome-CDS balances completeness and cost-efficiency.

An additional advantage of all three designs, is the ease of customization. Even in the exome-plus there is still room for ≈50 Mb of target regions to be added. For example, the few non-targeted RefSeq Genes and/or mRNA regions mentioned earlier or a new update in the non-coding RNA repertoire might be of interest. The regions uniquely identified by TransDecoder might also be added. BED files containing these regions are available on request.

## Conclusion

This study describes the development of two new target enrichment designs and the performance of the exome-plus. At a minimum sequencing depth of five, around 80% of the regions were covered completely and well over 90% of the base pairs were covered with a high reproducibility. In addition, a large number of variants were detected. Based on the results of the exome-plus, we estimate the performance of the exome-CDS. Together with the exome-1.0, these designs provide flexible solutions for a variety of research questions.

## Methods

Sample collection. Sixteen canine Labrador Retriever blood samples were obtained from a canine blood bank available at Ghent University to study genetic disorders^10^. Approval was granted by the local ethical (Faculty of Veterinary Medicine, Ghent University, Belgium) and deontological (Federal Public Service Health, Food Chain Safety and Environment, Brussels, Belgium) committees (EC2013_193). All experiments were carried out in accordance with the approved guidelines.

Design. For the exome-plus, from the University Of California Santa Cruz (UCSC) (http://genome.ucsc.edu/) table browser (Dog, CanFam3.1), the Ensembl Genes were selected from the Genes and Gene prediction tracks^11^,^12^. The output format was a BED file with the setting “exons (plus 0 bases at each end)”. Micro RNA sequence positions were downloaded from miRBase^3^. These files were combined with the protein coding genes, antisense transcripts and long non-coding transcripts and unpublished data on long non-coding transcripts^13^. Regions were merged using bedtools version v2.17.0. The total size of the design was 151,698,592 bp (≈6% of the genome) divided over 242,914 regions. For the exome-CDS, all files were identical except for the Ensembl Genes and the protein coding genes. From these 2 files, the CDS were predicted with TransDecoder^4^ and selected. The total size of the exome-CDS is 71,254,801 bp (≈3% of the genome) divided over 244,543 regions. Both BED files are available on request.

Roche Nimblegen WES enrichment assay. Our design was processed by the Roche Nimblegen custom design group (Madison, USA). Using an SSAHA algorithm, capturing baits were developed based on our design and the reference genome of the dog (*Canis familiaris* 3.1). Design settings for the baits allowed five or fewer single-base insertions, deletions or substitutions between the baits and the genome. Each bait was allowed to match at maximum up to 20 close matches in the genome. Regions under 100 bp were padded to 100 bp to increase capturing efficiency. After approval, the baits were generated and provided as SeqCap Developer Library.

DNA extraction. Genomic DNA was extracted with the DNeasy Blood & Tissue Kit (QIAGEN) with 100 μl of blood as input. The standard protocol was followed (including the RNAse A step) with the exception of the final elution step: instead of using 200 μl of Buffer AE, only 100 μl was used. The eluate was used again to elute a second and third time to increase the concentration. The DNA yield was measured with Quant-iT^TM^ Picogreen® dsDNA Assay (Life Technologies).

Sample preparation and sequencing. Extracted DNA was fragmented on a Covaris S2 System in a 130 μl volume (aim: 400 bp fragments, settings: duty cycle: 10%, intensity: 4, cycles per burst: 200, time: 55s). After shearing, another picogreen assay was performed. Depending on the yield after DNA-extraction, between 500 ng and 1 μg of the fragmented DNA was used as input for the library preparation. Samples were end repaired, A-tailed and ligated with TruSeq adapters using the reagents from the NEBNext Ultra DNA Library Prep Master mix set for Illumina (New England Biolabs) according to the manufacturer’s protocol. Size selection was performed on a 2% E-Gel (Invitrogen Life Technologies) (G4010-02), fragments were selected with an insert size around 300 bp. One μl of the ligated product was subsequently amplified in an enrichment PCR (10 cycles) for library quality assessment as recommended in the ‘SeqCap EZ Library SR User’s Guide’ (Nimblegen, Roche). Thereafter, the pre-capture LM-PCR was performed on the samples for 8 cycles as prescribed in the SeqCap EZ library protocol. The concentration of each PCR product was determined using Quant-iT^TM^ Picogreen® dsDNA Assay (Life Technologies). Four times four samples were equimolarly pooled to obtain a total DNA input of 1250 ng. The pooled library was hybridized for 67-68 hours with the baits (SeqCap Developer Library). The hybridized library was washed and the captured and pooled DNA was recovered. After a final amplification (LM-PCR, 18 cycles), the quality of the library was checked using the High Sensitivity DNA chip (Agilent).

QPCR. To check the fold enrichment after capturing, a qPCR is performed as a quality control step before sequencing. Five primer pairs were used, as described previously^1^. An additional qPCR was performed to determine the quantity of the library to ensure optimal cluster densities.

Sequencing. Each pool was sequenced in a separate run on the NextSeq 500 PE 75 bp.

Data-analysis. Data-analysis was performed using the CLC Genomics Workbench (Version 7.5.1, CLC Bio, Aarhus, Denmark). Data were trimmed with the following settings: ambiguous trim = no, quality trim = yes, quality limit = 0.05, use colorspace = no, create report = yes, also search on reversed sequence = yes, save discarded sequences = yes, remove 5′ terminal nucleotides = no, discard short reads = no, discard long reads = no, remove 3′ terminal nucleotides = no, trim adapter list = adapter list Illumina, save broken pairs = yes. The reference genome was downloaded from the UCSC genome browser^12^. For read mapping, the following parameters were used: mismatch cost = 2, insertion and deletion cost = 3, length fraction: 0.5, similarity fraction = 0.8, global alignment = no, auto-detect paired distances = yes, non-specific match handling = ignore, output mode = create reads track, create report = yes, collect un-mapped reads = yes, color space alignment = no, masking mode = no masking. Duplicated reads were removed with the Duplicate Mapped Reads Removal (Version 1.0 beta 6) plugin (setting: maximum representation of minority sequence (percent) to 20.0), create a second output file to save the removed reads = yes. Reads were locally realigned with the following settings: realign unaligned ends = yes, multi-pass realignment = 3, guidance-variant track = not set, force realignment to guidance variants = no, output mode = create reads track, output track of realigned regions = yes. Variants were called using fixed ploidy variant detection with the following settings: ploidy = 2, required variant probability = 90.0, ignore positions with coverage above = 100000, minimum coverage = 5, minimum count = 2, minimum frequency = 20.0%, restrict calling to target regions = no, ignore broken pairs = yes, ignore non-specific matches = reads, minimum read length = 20, base quality filter = no, relative read direction filter = yes (significance 1.0%), remove pyro-error variants = no, create track = yes, create table = yes, variant report = yes.

## Additional Information

**How to cite this article**: Broeckx, B. J.G. *et al.* Improved canine exome designs, featuring ncRNAs and increased coverage of protein coding genes. *Sci. Rep.* **5**, 12810; doi: 10.1038/srep12810 (2015).

## Supplementary Material

Supplementary Table S1

## Figures and Tables

**Table 1:** Statistics for exome sequencing sixteen dogs.

Sample	Pool	Total reads	Mapped reads	Duplicate reads	Remaining reads	Remaining (%)	Sequencing depth (x)
1	1	284,357,886	264,735,195	9,414,179	255,321,016	89.8	85.8
2	1	281,522,490	261,170,320	9,366,318	251,804,002	89.4	84.3
3	1	249,659,670	231,433,861	7,819,714	223,614,147	89.6	75.4
4	1	181,728,820	168,679,105	4,382,284	164,296,821	90.4	55.5
5	2	266,996,086	251,028,902	17,002,907	234,025,995	87.7	75.3
6	2	187,857,302	176,207,940	12,226,544	163,981,396	87.3	53.9
7	2	233,403,500	216,361,182	13,330,685	203,030,497	87.0	65.0
8	2	314,005,584	289,641,450	23,514,154	266,127,296	84.8	82.9
9	3	262,726,150	246,019,167	14,919,187	231,099,980	88.0	74.8
10	3	181,120,464	169,294,819	10,140,076	159,154,743	87.9	51.6
11	3	269,017,896	247,287,291	16,377,215	230,910,076	85.8	73.1
12	3	243,350,554	227,421,662	12,820,659	214,601,003	88.2	69.5
13	4	154,004,914	142,631,944	9,095,086	133,536,858	86.7	42.6
14	4	193,942,804	175,552,484	13,670,936	161,881,548	83.5	50.1
15	4	221,094,842	204,380,382	15,086,795	189,293,587	85.6	59.5
16	4	364,079,702	337,983,155	31,679,889	306,303,266	84.1	93.9

**Table 2:** Regions with a sequencing depth below 5x.

Sample	Pool	Regions with minimum sequencing depth <5x (%)	Regions with maximum sequencing depth < 5x (%)
1	1	42,705 (17.6)	6,977 (2.9)
2	1	42,749 (17.6)	6,980 (2.9)
3	1	45,739 (18.8)	7,307 (3.0)
4	1	55,643 (22.9)	9,346 (3.8)
5	2	41,798 (17.2)	8,502 (3.5)
6	2	54,390 (22.4)	10,953 (4.5)
7	2	50,032 (20.6)	10,439 (4.3)
8	2	40,793 (16.8)	8,238 (3.4)
9	3	44,312 (18.2)	8,884 (3.7)
10	3	57,615 (23.7)	11,185 (4.6)
11	3	44,956 (18.5)	8,698 (3.6)
12	3	46,948 (19.3)	9,125 (3.8)
13	4	66,381 (27.3)	12,380 (5.1)
14	4	57,903 (23.8)	10,446 (4.3)
15	4	41,031 (16.9)	7,457 (3.1)
16	4	54,075 (22.3)	10,156 (4.2)

**Table 3:** Coverage of targeted base pairs (≥5x).

Sample	Pool	base pairs exome-plus (%)	base pairs exome-CDS (%)
1	1	145,066,553 (95.6)	67,225,626 (94.3)
2	1	145,081,909 (95.6)	67,250,020 (94.4)
3	1	144,607,207 (95.3)	67,007,231 (94.0)
4	1	143,036,351 (94.3)	66,060,290 (92.7)
5	2	145,147,282 (95.7)	67,070,797 (94.1)
6	2	143,617,018 (94.7)	66,121,333 (92.8)
7	2	144,051,293 (95.0)	66,351,108 (93.1)
8	2	145,267,122 (95.8)	67,097,056 (94.2)
9	3	144,802,496 (95.5)	66,904,165 (93.9)
10	3	143,126,270 (94.3)	65,865,667 (92.4)
11	3	144,861,681 (95.5)	66,904,165 (93.9)
12	3	144,585,713 (95.3)	66,786,409 (93.7)
13	4	142,170,547 (93.7)	65,328,731 (91.7)
14	4	143,170,286 (94.4)	66,018,207 (92.7)
15	4	145,360,509 (95.8)	67,273,067 (94.4)
16	4	143,449,544 (94.6)	66,120,048 (92.8)

**Table 4:** Variants called inside the target regions.

Sample	Pool	Exome-plus (n)	Exome-CDS (n)
1	1	266,334	118,686
2	1	267,695	119,322
3	1	259,499	115,874
4	1	250,196	110,047
5	2	271,834	118,987
6	2	269,445	117,882
7	2	269,995	118,085
8	2	273,081	119,495
9	3	278,462	122,429
10	3	274,222	119,880
11	3	278,688	122,285
12	3	262,793	116,038
13	4	254,919	111,805
14	4	260,454	114,874
15	4	269,313	118,527
16	4	262,786	114,849

**Table 5:** Performance parameters of the exome-plus, the exome-CDS and the exome-1.0.

	exome-plus	exome-CDS	exome-1.0
fully covered regions (%)	79.7	85.4	84.9
base pairs covered (%)	95.1	93.4	90.2
% on target (%)	75.8	—	90.4
reproducibility regions (%)	63.5	71.4	79.9
reproducibility base pairs (%)	90.4	87.7	87.4
non-reference variants (n)	266,857	117,442	61,820
