# Supplementary material for: Improved canine exome designs, featuring ncRNAs and increased coverage of protein coding genes
Source: Sci Rep. 2015 Aug 3;5:12810. doi: 10.1038/srep12810 (PMC4522663; doi:10.1038/srep12810)
Supplement: Supplementary Table S1 [file srep12810-s1.pdf]

SUBJECT AREAS: GENETICS COMPUTATIONAL BIOLOGY AND BIOINFORMATICS

Correspondence and requests for materials should be addressed to D.D. (dieter.deforce@ugent.be).

Improved canine exome designs, featuring ncRNAs and increased coverage of protein coding genes

Bart J.G. Broeckx[1], Christophe Hitte[2], Frank Coopman[3], Geert E.C. Verhoeven[4], Sarah De Keulenaer[1], Ellen De Meester[1], Thomas Derrien[2], Jessica Alfoldi[5], Kerstin Lindblad-Toh[5,7], Tim Bosmans[6], Ingrid Gielen[4], Henri Van Bree[4], Bernadette Van Ryssen[4], Jimmy H. Saunders[4], Filip Van Nieuwerburgh[1‡], Dieter Deforce[1‡*]

[1] Laboratory of Pharmaceutical Biotechnology, Faculty of Pharmaceutical Sciences, Ghent University, Ghent, Belgium.

[2] Institut de Génétique et Développement de Rennes, CNRS-URM6290, Université Rennes1, Rennes, France.

[3] Department of Applied Biosciences, Faculty of Biosciences Engineering, Ghent University, Ghent, Belgium.

[4] Department of Medical Imaging and Small Animal Orthopaedics, Faculty of Veterinary Medicine, Ghent University, Merelbeke, Belgium.

[5] Broad Institute of MIT and Harvard, Cambridge, Massachusetts, USA.

[6] Department of Medicine and Clinical Biology of Small Animals, Faculty of Veterinary Medicine, Ghent University, Merelbeke, Belgium.

[7] Science for Life Laboratory, Department of Medical Biochemistry and Microbiology, Uppsala University, Uppsala, Sweden.

‡ These authors contributed equally to this work.

Supplementary Table S1 Per sample per chromosome percentage reads on target (calculated as mapped reads on target/total number of mapped reads)

Chr	1	2	3	4	5	6	7	8	9	10	11	12	13	14	15	16
1	81.0	80.6	80.8	81.0	77.6	77.4	76.9	77.4	77.5	77.7	77.2	77.2	77.1	77.1	78.0	77.7
2	80.5	80.1	80.2	80.1	76.7	76.6	76.0	76.8	77.1	77.0	76.8	76.7	76.4	76.6	77.6	77.2
3	78.2	77.9	78.2	78.1	74.4	74.2	73.5	74.1	74.3	74.4	73.9	74.0	73.8	73.5	74.6	74.4
4	80.5	80.2	80.5	80.3	77.2	76.9	76.3	77.0	77.1	77.3	76.8	76.7	76.6	76.5	77.7	77.3
5	82.0	82.0	82.3	82.1	79.2	78.8	78.1	79.1	79.2	79.2	79.1	79.0	78.7	78.6	79.8	79.3
6	81.1	80.8	81.2	80.9	77.3	77.1	76.4	77.0	77.2	77.1	76.8	77.1	76.8	76.7	77.8	77.4
7	80.7	80.9	82.0	79.6	76.5	76.0	74.9	76.0	75.8	76.2	76.0	76.2	75.2	74.8	76.6	75.7
8	79.5	79.3	79.6	79.3	76.2	75.8	74.9	75.8	75.9	76.0	75.7	75.9	75.6	75.4	76.5	76.3
9	84.5	84.2	84.6	84.4	81.4	81.0	80.6	81.5	81.5	81.5	81.3	81.4	81.2	81.5	82.4	82.0
10	81.9	81.6	81.9	81.7	78.7	78.4	77.7	78.5	78.7	78.8	78.4	78.4	78.2	78.3	79.3	78.9
11	79.4	79.2	79.4	79.5	76.2	75.9	75.2	75.9	76.2	76.2	75.8	75.9	75.7	75.6	76.7	76.3
12	80.2	79.9	80.2	80.0	76.7	76.5	75.6	76.4	76.6	76.8	76.2	76.3	76.1	76.2	77.1	76.8
13	75.3	75.0	74.9	77.8	74.3	74.1	73.2	74.1	74.2	74.2	73.9	73.9	73.7	73.6	74.8	74.4
14	78.3	78.0	78.5	78.1	74.6	74.3	73.5	74.2	74.4	74.5	73.9	74.0	73.7	73.7	74.8	74.6
15	80.6	80.3	80.6	80.4	76.9	76.6	75.9	76.8	77.0	77.1	76.6	76.6	76.3	76.3	77.6	77.2
16	74.9	74.6	74.6	74.7	71.1	70.8	70.0	71.0	71.3	70.9	70.5	70.7	70.9	70.6	71.8	70.6
17	80.9	80.9	80.6	80.4	77.4	77.0	76.5	77.2	77.7	77.7	77.2	77.2	77.0	77.1	78.2	77.5
18	80.2	80.0	80.3	79.8	77.1	76.6	75.8	76.6	76.8	76.9	76.5	76.6	76.5	76.6	77.3	77.0
19	72.0	71.3	72.4	71.7	68.4	68.3	66.6	67.7	67.5	67.8	67.5	67.4	67.2	66.5	68.4	67.9
20	82.7	82.4	82.7	82.4	79.6	79.2	78.4	79.4	79.5	79.7	79.3	79.5	79.2	79.5	80.1	79.7
21	78.0	77.6	77.6	77.3	74.4	74.0	73.4	74.2	74.4	74.5	74.2	74.3	73.8	73.7	74.8	73.5
22	73.8	73.4	73.8	73.6	70.0	70.0	69.1	69.4	69.6	69.9	69.1	69.4	69.2	68.9	69.9	69.7
23	80.2	80.0	80.3	80.0	76.6	76.2	75.6	76.3	76.5	76.7	76.2	76.3	76.0	75.9	77.0	76.6
24	81.1	81.0	81.4	81.2	77.7	77.5	77.0	77.7	77.9	78.2	77.7	77.8	77.7	77.8	78.8	78.3
25	79.1	78.9	79.3	79.1	75.7	75.4	74.7	75.5	75.7	75.8	75.5	75.4	74.9	74.9	76.1	75.7
26	81.3	80.9	81.3	81.1	77.7	77.5	77.1	77.8	77.8	78.0	77.8	77.8	77.7	77.5	78.6	78.2
27	81.4	81.1	81.3	81.0	77.5	77.1	76.5	77.3	77.5	77.5	77.2	77.2	77.1	77.2	78.2	77.8
28	81.4	81.0	81.4	81.1	77.7	77.3	76.8	77.7	77.7	77.8	77.6	77.5	77.0	77.1	78.6	78.0
29	76.5	76.1	76.4	76.3	72.3	72.0	71.3	72.1	72.1	72.3	71.6	71.7	71.5	71.2	72.5	72.5
30	82.4	82.1	82.6	82.1	78.6	78.1	77.6	78.4	78.7	78.7	78.3	78.3	78.1	78.3	79.5	79.0
31	70.3	69.8	69.6	69.4	65.3	65.1	64.4	64.8	64.9	64.9	64.2	63.5	64.5	63.8	64.8	64.4
32	70.7	69.8	70.3	69.6	62.6	62.7	63.4	63.4	63.2	62.1	62.4	60.5	60.9	59.4	62.9	62.9
33	80.6	80.4	80.7	80.4	76.9	76.5	75.8	76.7	76.8	76.8	76.5	76.5	76.1	76.2	77.6	77.1
34	77.7	77.4	77.6	77.4	73.4	73.2	72.7	73.2	73.2	73.2	72.9	72.8	72.5	72.0	73.6	73.2
35	80.5	80.2	80.4	80.4	77.1	77.0	76.3	76.9	77.1	77.1	76.8	76.9	76.4	76.4	77.4	77.1
36	80.6	80.3	80.7	80.3	76.7	76.2	75.5	76.7	76.7	76.7	76.4	76.2	75.8	76.2	77.7	77.1
37	80.3	80.0	80.2	79.9	75.8	75.5	74.9	75.5	75.3	75.5	75.1	75.3	75.2	75.0	76.2	75.9
38	78.3	77.9	78.2	78.0	74.9	74.5	73.3	74.1	74.5	74.5	74.0	74.2	73.8	74.2	74.6	74.4
X	66.5	66.1	67.8	67.7	63.0	62.9	60.8	61.7	61.9	62.0	62.5	61.6	61.1	61.2	62.6	62.7
